# Crystal structure of bis­[4-(di­methyl­amino)­pyridinium] aqua­bis­(oxalato)oxidovanadate(IV) dihydrate

**DOI:** 10.1107/S2056989016009695

**Published:** 2016-06-21

**Authors:** Hiba Sehimi, Ichraf Chérif, Mohamed Faouzi Zid

**Affiliations:** a Laboratoire de Matériaux et Cristallochimie, Faculté des Sciences de Tunis, Université de Tunis El Manar, 2092 Manar II Tunis, Tunisia; b Université de Gabès, Faculté des Sciences de Gabès, Campus Universitaire, Cité Erriadh Zrig, Gabès, 6072, Tunisia

**Keywords:** crystal structure, 4-(di­methyl­amino)­pyridine, π–π inter­actions, vanadium(IV), oxalate ligand

## Abstract

The title organic–inorganic hybrid salt, (C_7_H_11_N_2_)_2_[V(C_2_O_4_)_2_O(H_2_O)]·2H_2_O, shows a distorted octa­hedral coordination environment for the vanadium(IV) atom in the anion (point group symmetry 2), with four O atoms from two symmetry-related chelating oxalate dianions and two O atoms in *trans* configuration from a coordinating water mol­ecule and a terminal vanadyl O atom. In the crystal, (001) layers of cations and anions alternate along [001]. The anionic layers are built up by inter­molecular O—H⋯O hydrogen bonds involving the coordinating and solvent water mol­ecules. The cationic layers are linked to the anionic layers *via* N—H⋯O hydrogen bonds between the pyridinium group and the non-coordinating O atoms of the oxalate group. The 4-(di­methyl­amino)­pyridinium cations are also engaged in π–π stacking with their anti­parallel neighbours [centroid-to-centroid distance = 3.686 (2) Å]. Considering all supra­molecular features, a three-dimensional network structure is accomplished.

## Abstract 2

The vanadium(IV) atom in the title compound is located on a twofold rotation axis and has a distorted octa­hedral coordination sphere made up from two symmetry-related oxalate ligands, one vanadyl O atom and a water mol­ecule.

## Chemical context   

Because of the great importance of vanadium as an effective metal anti­tumor agent (Evangelou, 2002 ▸) and the vanadyl anti­diabetic factor *via* its manifested insulin-mimetic activity (Goc, 2006 ▸), the coordination chemistry of this element has received much attention over the past years through the design and synthesis of organic–inorganic hybrid salts and the investigation of their solution chemistry. In addition to that, the use of pyridine and its derivatives in those hybrid materials may also provide biological activity as reported by Markees *et al.* (1968 ▸). Many compounds containing the vanadyl V=O group combined with oxalate ligands have been isolated as mononuclear (Lin *et al.*, 2004 ▸; Aghabozorg *et al.*, 2007 ▸; Oughtred *et al.*, 1976 ▸) or dinuclear (Zheng *et al.*, 1998 ▸) compounds.
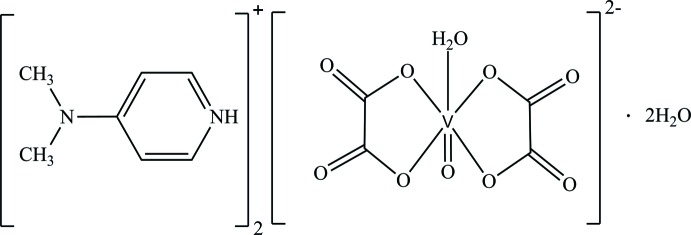


In this context, we report on the synthesis and crystal structure of the title organic–inorganic hybrid salt, (C_7_H_11_N_2_)_2_[V(C_2_O_4_)_2_O(H_2_O)]·2H_2_O, (I).

## Structural commentary   

**Figure 1:**
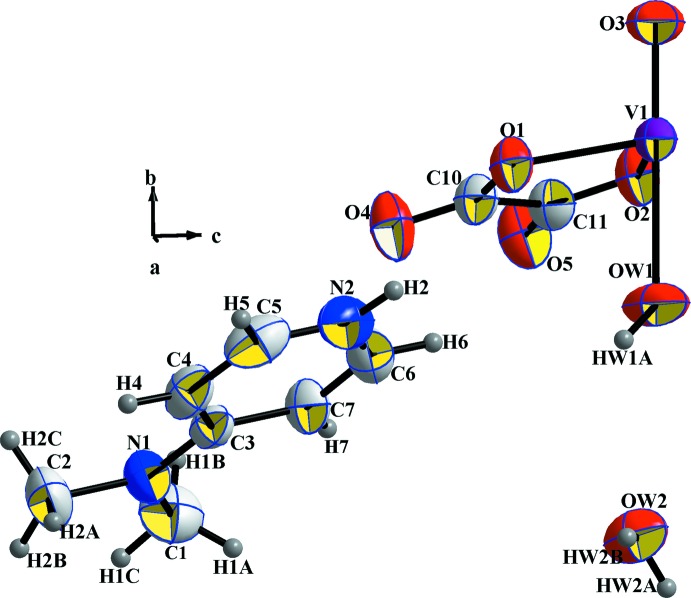
The asymmetric unit of (I) showing the atom-numbering scheme. Displacement ellipsoids are drawn at the 50% probability level for non-H atoms.

The vanadium atom V1, the double-bonded oxygen atom O3 of the vanadyl group and the oxygen atom of the coordinating water mol­ecule O*W*1 lie on a twofold rotation axis. Thus, the asymmetric unit of the title compound corresponds to half of the mol­ecular formula which consequently contains one half-anionic complex [V_1/2_(C_2_O_4_)O_1/2_(HO_1/2_)]^−^, one 4-(di­methyl­amino)­pyridinium cation (C_7_H_11_N_2_)^+^ protonated at the N2 atom of the heterocyclic ring, and one solvent water mol­ecule (Fig. 1 ▸). The anionic complex has an overall charge of 2−, requiring a vanadium atom with an oxidation state of +IV. This formal value is in good agreement with the bond-valence-sum calculation (Brown & Altermatt, 1985 ▸), resulting in a value of 4.20 (3) valence units.

The V^IV^ ion is coordinated by four oxygen atoms of two symmetry-related chelating oxalate dianions, defining the equatorial plane, and two axial oxygen atoms from a water mol­ecule and the vanadyl oxygen atom. The resulting octa­hedral coordination sphere is considerably distorted. The V—O_oxalate_ bond lengths (Table 1 ▸) are in good agreement with structures containing the same [V(C_2_O_4_)_2_O(H_2_O)]^2−^ anion and di­ammonium (Oughtred *et al.*, 1976 ▸) or piperazinium (Lin *et al.*, 2004 ▸) as counter-cations. The short V1=O3 distance of 1.600 (3) Å is typical for a double-bonded vanadyl group and the longest V—O bond involves the aqua ligand, again in agreement with the structures of the related compounds with different cations. The shortest distances between vanadium atoms in the isolated complexes are equal to 7.689 (4) Å along [010] (corresponding to the length of the *b* axis) and 8.287 (1) Å along [010], while a shorter distance equal to 5.176 (5) Å along the [001] direction is reported by Aghabozorg *et al.* (2007 ▸) for the related piperazinium compound. The oxalate anion is planar (root-mean-deviation of fitted atoms = 0.0343 Å); the two symmetry-related oxalate ligands subtend a dihedral angle of 32.59 (4)° between the least-squares planes. The slightly elongated C—C bond length of 1.552 (3) Å in the oxalate anion is in agreement with the value of 1.539 (2) Å reported for other oxalate complexes (Belaj *et al.*, 2000 ▸). Bond lengths and angles of the 4-(di­methyl­amino)­pyridinium cation are consistent with those found in salts with the same cationic entity (Ben Nasr *et al.*, 2015 ▸) with C—N distances in the range 1.326 (3)–1.458 (3) Å and C—C distances between 1.343 (3) and 1.413 (3) Å.

## Supra­molecular features   

**Figure 2:**
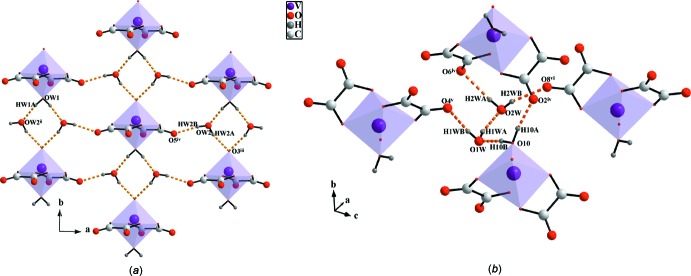
View of O—H⋯O hydrogen bonds (orange dashed lines) developed by both coordinating and non-coordinating water mol­ecules in (*a*) the title compound [symmetry codes: (ii) *x* − 1, *y*, *z*; (iii) *x* + 1, *y* − 1, *z*; (iv) *x* + 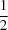, *y* − 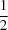, *z*] and (*b*) the compound (C_4_H_12_N_2_)[V(C_2_O_4_)_2_O(H_2_O)]·2H_2_O [symmetry codes: (iv) −*x* + 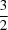, *y* + 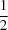, −*z* + 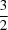; (v) −*x* + 1, −*y* + 2, −*z* + 1; (vi) −*x* + 2, −*y* + 2, −*z* + 2.]

**Figure 3:**
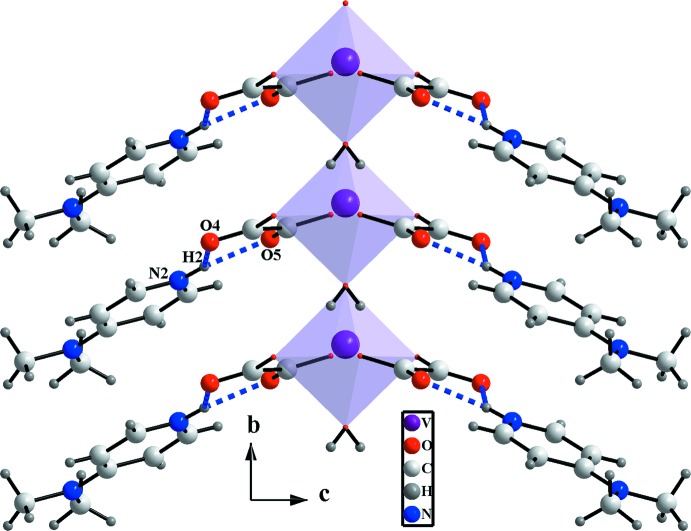
View of the N—H⋯O hydrogen bonds (blue dashed lines) developed between anionic and cationic entities.

**Figure 4:**
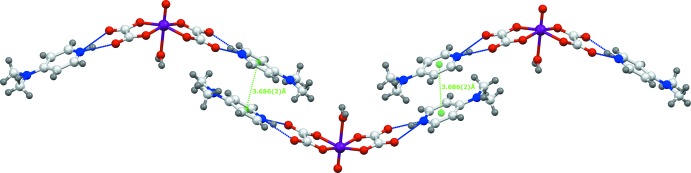
π–π stacking inter­actions (green dashed lines) between adjacent anti-parallel organic cations, forming zigzag chains.

**Figure 5:**
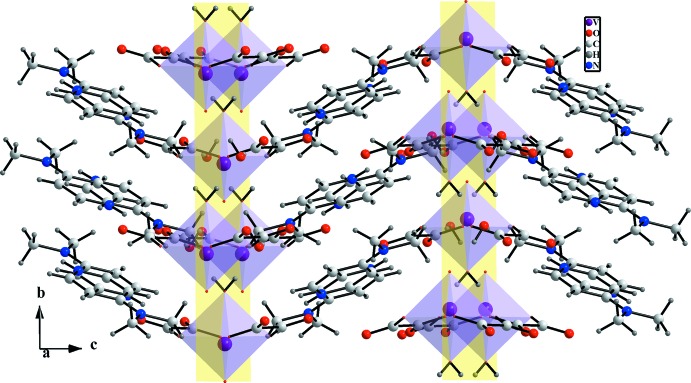
View of the structure packing of (I) showing anionic layers (yellow planes), zigzag chains and π–π stacking.

Within the crystal packing, all components are connected by an extensive hydrogen-bonding network (Table 2 ▸). The cations and anions are aligned into layers parallel to (001). O—H⋯O hydrogen bonds involving the coordinating O*W*1 water mol­ecule as donor group and the solvent O*W*2 mol­ecule as both acceptor and donor groups consolidate the anionic layers parallel to (001), as shown in Fig. 2 ▸ *a*. In the structure of the related piperazinium compound (Aghabozorg *et al.*, 2007 ▸), a more complex three-dimensional arrangement of the O—H⋯O hydrogen bonds is realized (Fig. 2 ▸ *b*). Along the [001] direction, N—H⋯O hydrogen bonds involving the proton­ated N2 atom of the 4-(di­methyl­amino)­pyridinium cation as double-donor group and non-coordinating O atoms of the oxalate dianion as acceptors ensure the connection between the anionic and cationic layers in the title structure, as shown in Fig. 3 ▸. Furthermore, π–π stacking inter­actions between anti­parallel-arranged pyridinium rings [centroid-to-centroid distance = 3.686 (2) Å; Fig. 4 ▸] are present and consolidate the three-dimensional network (Fig. 5 ▸).

## Synthesis and crystallization   

A solution of 0.5 mmol of vanadium(V) pentoxide dissolved in 10 cm^3^ of distilled water was added to a solution of 1 mmol of oxalic acid dissolved in 10 cm^3^ of distilled water. Then, a solution of 1 mmol of 4-(di­methyl­amino)­pyridine dissolved in 10 cm^3^ of distilled water was poured slowly until pH ≃ 4. The obtained blue solution was placed in a petri dish at room temperature for almost one month until purple crystals suitable for a structural study appeared.

## Refinement   

Crystal data, data collection and structure refinement details are summarized in Table 3 ▸. H atoms bonded to C and N atoms were placed at geometrically calculated positions using a riding model. C—H distances were fixed at 0.93 Å for aromatic H atoms and 0.96 Å for methyl H atoms, with *U*_iso_(H) = 1.2*U*_eq_(C_aromatic_) or 1.5*U*_eq_(C_meth­yl_). The N—H distance was fixed at 0.86 Å. All water H atoms were located from a difference-Fourier map and were refined with restraints [O—H 0.85 (1) Å; H⋯H 1.387 (1) Å].

## Supplementary Material

Crystal structure: contains datablock(s) I. DOI: 10.1107/S2056989016009695/wm5298sup1.cif

Structure factors: contains datablock(s) I. DOI: 10.1107/S2056989016009695/wm5298Isup2.hkl

CCDC reference: 1485722

Additional supporting information: crystallographic information; 3D view; checkCIF report

## Figures and Tables

**Table 1:** Selected bond lengths (Å)

V1—O3	1.600 (3)	V1—O1	1.997 (1)
V1—O2^i^	1.986 (2)	V1—O1^i^	1.997 (1)
V1—O2	1.986 (2)	V1—O*W*1	2.262 (3)

Symmetry code: (i) 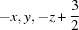.

**Table 2:** Hydrogen-bond geometry (Å, °)

*D*—H⋯*A*	*D*—H	H⋯*A*	*D*⋯*A*	*D*—H⋯*A*
O*W*1—H*W*1*A*⋯O*W*2^ii^	0.84 (1)	1.90 (1)	2.740 (3)	172 (3)
O*W*2—H*W*2*A*⋯O3^iii^	0.86 (1)	1.95 (1)	2.792 (3)	166 (2)
O*W*2—H*W*2*B*⋯O5^iv^	0.85 (1)	1.96 (1)	2.779 (2)	161 (3)
N2—H2⋯O4	0.86	2.32	3.002 (3)	136
N2—H2⋯O5	0.86	2.02	2.777 (3)	146

Symmetry codes: (ii) 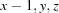; (iii) 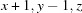; (iv) 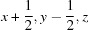.

**Table 3:** Experimental details

Crystal data	
Chemical formula	(C_7_H_11_N_2_)_2_[V(C_2_O_4_)_2_O(H_2_O)]·2H_2_O
*M*_r_	543.38
Crystal system, space group	Monoclinic, *C*2/*c*
Temperature (K)	298
*a*, *b*, *c* (Å)	14.682 (2), 7.689 (4), 21.280 (3)
β (°)	97.197 (10)
*V* (Å^3^)	2383.3 (13)
*Z*	4
Radiation type	Mo *K*α
μ (mm^−1^)	0.49
Crystal size (mm)	0.46 × 0.28 × 0.21

Data collection	
Diffractometer	Enraf–Nonius CAD-4
Absorption correction	ψ scan (North *et al.*, 1968 ▸)
*T*_min_, *T*_max_	0.841, 0.908
No. of measured, independent and observed [*I* > 2σ(*I*)] reflections	4165, 2599, 1850
*R*_int_	0.028
(sin θ/λ)_max_ (Å^−1^)	0.638

Refinement	
*R*[*F* ^2^ > 2σ(*F* ^2^)], *wR*(*F* ^2^), *S*	0.038, 0.106, 1.01
No. of reflections	2599
No. of parameters	174
No. of restraints	4
H-atom treatment	H atoms treated by a mixture of independent and constrained refinement
Δρ_max_, Δρ_min_ (e Å^−3^)	0.25, −0.26

Computer programs: *CAD-4 EXPRESS* (Duisenberg, 1992 ▸), *XCAD4* (Harms & Wocadlo, 1995 ▸), *SHELXS97* (Sheldrick, 2008 ▸), *SHELXL2014* (Sheldrick, 2015 ▸), *DIAMOND* (Brandenburg, 2006 ▸), *WinGX* (Farrugia, 2012 ▸) and *publCIF* (Westrip, 2010 ▸).

## supplementary crystallographic information

### Crystal data

**Table:**

(C_7_H_11_N_2_)_2_[V(C_2_O_4_)_2_O(H_2_O)]·2H_2_O	*F*(000) = 1132
*M**_r_* = 543.38	*D*_x_ = 1.514 Mg m^−^^3^
Monoclinic, *C*2/*c*	Mo *K*α radiation, λ = 0.71073 Å
*a* = 14.682 (2) Å	Cell parameters from 25 reflections
*b* = 7.689 (4) Å	θ = 10–15°
*c* = 21.280 (3) Å	µ = 0.49 mm^−^^1^
β = 97.197 (10)°	*T* = 298 K
*V* = 2383.3 (13) Å^3^	Prism, purple
*Z* = 4	0.46 × 0.28 × 0.21 mm

### Data collection

**Table:**

Enraf–Nonius CAD-4 diffractometer	*R*_int_ = 0.028
Radiation source: fine-focus sealed tube	θ_max_ = 27.0°, θ_min_ = 2.8°
ω/2θ scans	*h* = −18→5
Absorption correction: ψ scan (North *et al.*, 1968)	*k* = −1→9
*T*_min_ = 0.841, *T*_max_ = 0.908	*l* = −27→27
4165 measured reflections	2 standard reflections every 120 reflections
2599 independent reflections	intensity decay: 1.4%
1850 reflections with *I* > 2σ(*I*)	

### Refinement

**Table:**

Refinement on *F*^2^	Primary atom site location: heavy-atom method
Least-squares matrix: full	Secondary atom site location: difference Fourier map
*R*[*F*^2^ > 2σ(*F*^2^)] = 0.038	Hydrogen site location: mixed
*wR*(*F*^2^) = 0.106	H atoms treated by a mixture of independent and constrained refinement
*S* = 1.01	*w* = 1/[σ^2^(*F*_o_^2^) + (0.0482*P*)^2^ + 1.2556*P*] where *P* = (*F*_o_^2^ + 2*F*_c_^2^)/3
2599 reflections	(Δ/σ)_max_ < 0.001
174 parameters	Δρ_max_ = 0.25 e Å^−^^3^
4 restraints	Δρ_min_ = −0.26 e Å^−^^3^

### Special details

**Table:**

Geometry. All esds (except the esd in the dihedral angle between two l.s. planes) are estimated using the full covariance matrix. The cell esds are taken into account individually in the estimation of esds in distances, angles and torsion angles; correlations between esds in cell parameters are only used when they are defined by crystal symmetry. An approximate (isotropic) treatment of cell esds is used for estimating esds involving l.s. planes.
Refinement. Refinement of F^2^ against ALL reflections. The weighted R-factor wR and goodness of fit S are based on F^2^, conventional R-factors R are based on F, with F set to zero for negative F^2^. The threshold expression of F^2^ > 2sigma(F^2^) is used only for calculating R-factors(gt) etc. and is not relevant to the choice of reflections for refinement. R-factors based on F^2^ are statistically about twice as large as those based on F, and R- factors based on ALL data will be even larger.

### Fractional atomic coordinates and isotropic or equivalent isotropic displacement parameters (Å^2^)

**Table:**

	*x*	*y*	*z*	*U*_iso_*/*U*_eq_	
V1	0.0000	0.84234 (7)	0.7500	0.03574 (16)	
O1	−0.02877 (10)	0.7977 (2)	0.65720 (7)	0.0446 (4)	
OW1	0.0000	0.5482 (3)	0.7500	0.0707 (9)	
O2	0.12736 (10)	0.8022 (2)	0.73095 (7)	0.0477 (4)	
O3	0.0000	1.0504 (3)	0.7500	0.0583 (7)	
O4	0.03921 (12)	0.7053 (3)	0.57518 (8)	0.0618 (5)	
O5	0.20402 (11)	0.7168 (3)	0.65270 (8)	0.0604 (5)	
C10	0.04062 (15)	0.7502 (3)	0.63017 (10)	0.0424 (5)	
C11	0.13301 (15)	0.7551 (3)	0.67444 (11)	0.0430 (5)	
HW1A	−0.0389 (16)	0.480 (3)	0.7310 (12)	0.072 (9)*	
OW2	0.88723 (13)	0.3042 (3)	0.68686 (11)	0.0639 (5)	
HW2A	0.9136 (14)	0.216 (2)	0.7059 (11)	0.056 (8)*	
HW2B	0.8295 (7)	0.290 (3)	0.6839 (15)	0.081 (10)*	
N1	0.34041 (15)	0.3258 (3)	0.39759 (9)	0.0522 (5)	
N2	0.21437 (16)	0.5684 (3)	0.53481 (11)	0.0596 (6)	
H2	0.1881	0.6207	0.5634	0.071*	
C1	0.4371 (2)	0.2774 (4)	0.40787 (15)	0.0690 (8)	
H1A	0.4496	0.2154	0.4472	0.103*	
H1B	0.4743	0.3805	0.4095	0.103*	
H1C	0.4512	0.2046	0.3737	0.103*	
C2	0.2904 (3)	0.2816 (4)	0.33610 (12)	0.0774 (10)	
H2A	0.2329	0.2286	0.3420	0.116*	
H2B	0.3261	0.2017	0.3146	0.116*	
H2C	0.2792	0.3853	0.3112	0.116*	
C3	0.29925 (15)	0.4045 (3)	0.44227 (10)	0.0387 (5)	
C4	0.20526 (16)	0.4499 (4)	0.43279 (12)	0.0520 (6)	
H4	0.1699	0.4248	0.3945	0.062*	
C5	0.16691 (18)	0.5297 (4)	0.47941 (14)	0.0609 (7)	
H5	0.1050	0.5586	0.4725	0.073*	
C6	0.30270 (19)	0.5261 (4)	0.54591 (12)	0.0558 (7)	
H6	0.3352	0.5526	0.5851	0.067*	
C7	0.34650 (15)	0.4462 (3)	0.50226 (10)	0.0463 (5)	
H7	0.4083	0.4181	0.5116	0.056*	

### Atomic displacement parameters (Å^2^)

**Table:**

	*U*^11^	*U*^22^	*U*^33^	*U*^12^	*U*^13^	*U*^23^
V1	0.0295 (3)	0.0374 (3)	0.0388 (3)	0.000	−0.0018 (2)	0.000
O1	0.0299 (7)	0.0617 (10)	0.0400 (8)	0.0049 (7)	−0.0041 (6)	−0.0010 (7)
OW1	0.0651 (18)	0.0359 (14)	0.098 (2)	0.000	−0.0396 (16)	0.000
O2	0.0287 (7)	0.0703 (12)	0.0423 (8)	−0.0025 (7)	−0.0032 (7)	−0.0066 (8)
O3	0.0657 (16)	0.0377 (13)	0.0698 (16)	0.000	0.0018 (13)	0.000
O4	0.0446 (10)	0.0979 (15)	0.0415 (9)	0.0084 (10)	−0.0004 (8)	−0.0118 (9)
O5	0.0311 (9)	0.0928 (14)	0.0571 (10)	0.0038 (9)	0.0048 (8)	−0.0131 (10)
C10	0.0340 (12)	0.0502 (14)	0.0414 (12)	0.0018 (10)	−0.0016 (9)	0.0011 (11)
C11	0.0310 (11)	0.0501 (14)	0.0467 (12)	−0.0023 (10)	−0.0003 (9)	−0.0008 (11)
OW2	0.0418 (10)	0.0568 (12)	0.0867 (14)	−0.0043 (9)	−0.0175 (10)	0.0098 (10)
N1	0.0532 (12)	0.0595 (13)	0.0433 (10)	0.0013 (11)	0.0041 (9)	−0.0075 (9)
N2	0.0658 (15)	0.0561 (14)	0.0621 (13)	0.0034 (12)	0.0287 (12)	−0.0001 (11)
C1	0.0564 (17)	0.0740 (19)	0.0806 (19)	0.0109 (15)	0.0245 (15)	−0.0059 (16)
C2	0.105 (3)	0.081 (2)	0.0440 (15)	−0.010 (2)	0.0019 (16)	−0.0155 (14)
C3	0.0365 (11)	0.0384 (11)	0.0399 (10)	−0.0019 (9)	−0.0001 (9)	0.0043 (9)
C4	0.0416 (13)	0.0595 (16)	0.0517 (13)	0.0012 (12)	−0.0068 (11)	0.0096 (12)
C5	0.0406 (14)	0.0618 (17)	0.0824 (19)	0.0131 (13)	0.0158 (14)	0.0190 (15)
C6	0.0608 (17)	0.0625 (17)	0.0440 (12)	−0.0085 (14)	0.0067 (12)	−0.0042 (12)
C7	0.0353 (12)	0.0585 (15)	0.0435 (11)	−0.0018 (11)	−0.0011 (9)	−0.0017 (11)

### Geometric parameters (Å, º)

**Table:**

V1—O3	1.600 (3)	N2—C5	1.326 (3)
V1—O2^i^	1.986 (2)	N2—C6	1.329 (3)
V1—O2	1.986 (2)	N2—H2	0.8600
V1—O1	1.997 (1)	C1—H1A	0.9600
V1—O1^i^	1.997 (1)	C1—H1B	0.9600
V1—OW1	2.262 (3)	C1—H1C	0.9600
O1—C10	1.284 (3)	C2—H2A	0.9600
OW1—HW1A	0.842 (10)	C2—H2B	0.9600
O2—C11	1.268 (3)	C2—H2C	0.9600
O4—C10	1.218 (3)	C3—C7	1.411 (3)
O5—C11	1.228 (3)	C3—C4	1.413 (3)
C10—C11	1.552 (3)	C4—C5	1.348 (4)
OW2—HW2A	0.855 (9)	C4—H4	0.9300
OW2—HW2B	0.848 (10)	C5—H5	0.9300
N1—C3	1.334 (3)	C6—C7	1.343 (3)
N1—C1	1.458 (3)	C6—H6	0.9300
N1—C2	1.458 (3)	C7—H7	0.9300
			
O3—V1—O2^i^	98.94 (6)	C5—N2—H2	120.2
O3—V1—O2	98.94 (6)	C6—N2—H2	120.2
O2^i^—V1—O2	162.13 (11)	N1—C1—H1A	109.5
O3—V1—O1	99.90 (5)	N1—C1—H1B	109.5
O2^i^—V1—O1	95.01 (6)	H1A—C1—H1B	109.5
O2—V1—O1	81.91 (6)	N1—C1—H1C	109.5
O3—V1—O1^i^	99.90 (5)	H1A—C1—H1C	109.5
O2^i^—V1—O1^i^	81.91 (6)	H1B—C1—H1C	109.5
O2—V1—O1^i^	95.01 (6)	N1—C2—H2A	109.5
O1—V1—O1^i^	160.21 (10)	N1—C2—H2B	109.5
O3—V1—OW1	180.0	H2A—C2—H2B	109.5
O2^i^—V1—OW1	81.06 (6)	N1—C2—H2C	109.5
O2—V1—OW1	81.06 (6)	H2A—C2—H2C	109.5
O1—V1—OW1	80.10 (5)	H2B—C2—H2C	109.5
O1^i^—V1—OW1	80.10 (5)	N1—C3—C7	122.2 (2)
C10—O1—V1	114.24 (13)	N1—C3—C4	122.1 (2)
V1—OW1—HW1A	128.7 (19)	C7—C3—C4	115.7 (2)
C11—O2—V1	114.38 (14)	C5—C4—C3	119.8 (2)
O4—C10—O1	126.3 (2)	C5—C4—H4	120.1
O4—C10—C11	119.9 (2)	C3—C4—H4	120.1
O1—C10—C11	113.77 (19)	N2—C5—C4	122.4 (2)
O5—C11—O2	125.8 (2)	N2—C5—H5	118.8
O5—C11—C10	118.9 (2)	C4—C5—H5	118.8
O2—C11—C10	115.2 (2)	N2—C6—C7	122.0 (2)
HW2A—OW2—HW2B	108.9 (15)	N2—C6—H6	119.0
C3—N1—C1	121.9 (2)	C7—C6—H6	119.0
C3—N1—C2	121.5 (2)	C6—C7—C3	120.4 (2)
C1—N1—C2	116.5 (2)	C6—C7—H7	119.8
C5—N2—C6	119.7 (2)	C3—C7—H7	119.8
			
V1—O1—C10—O4	175.1 (2)	C1—N1—C3—C4	−179.3 (2)
V1—O1—C10—C11	−5.5 (2)	C2—N1—C3—C4	−0.2 (4)
V1—O2—C11—O5	−177.1 (2)	N1—C3—C4—C5	−179.8 (2)
V1—O2—C11—C10	4.0 (3)	C7—C3—C4—C5	0.8 (4)
O4—C10—C11—O5	1.5 (4)	C6—N2—C5—C4	−0.9 (4)
O1—C10—C11—O5	−178.0 (2)	C3—C4—C5—N2	0.1 (4)
O4—C10—C11—O2	−179.5 (2)	C5—N2—C6—C7	0.8 (4)
O1—C10—C11—O2	1.1 (3)	N2—C6—C7—C3	0.1 (4)
C1—N1—C3—C7	0.1 (4)	N1—C3—C7—C6	179.7 (2)
C2—N1—C3—C7	179.2 (2)	C4—C3—C7—C6	−0.9 (4)

Symmetry code: (i) −*x*, *y*, −*z*+3/2.

### Hydrogen-bond geometry (Å, º)

**Table:**

*D*—H···*A*	*D*—H	H···*A*	*D*···*A*	*D*—H···*A*
O*W*1—H*W*1*A*···O*W*2^ii^	0.84 (1)	1.90 (1)	2.740 (3)	172 (3)
O*W*2—H*W*2*A*···O3^iii^	0.86 (1)	1.95 (1)	2.792 (3)	166 (2)
O*W*2—H*W*2*B*···O5^iv^	0.85 (1)	1.96 (1)	2.779 (2)	161 (3)
N2—H2···O4	0.86	2.32	3.002 (3)	136
N2—H2···O5	0.86	2.02	2.777 (3)	146

Symmetry codes: (ii) *x*−1, *y*, *z*; (iii) *x*+1, *y*−1, *z*; (iv) *x*+1/2, *y*−1/2, *z*.
